# Prevalence and risk factors of depression in college students in Northeast China during the COVID-19 pandemic: a cross-sectional study

**DOI:** 10.1186/s40359-025-03944-x

**Published:** 2026-01-07

**Authors:** Yanze Cui, Liying Yang, Wanqiu Yang, Xiaohong Wang, Jiazhou Liu, Yanqing Wang, Jiacheng Liu, Dan Leng, Borui Yang, Na Zhao, Chuanyi Kang

**Affiliations:** 1 Department of Scientific Research Management Office, The First Affiliated Hospital of Harbin Medical University, Harbin, Heilongjiang Province China; 2 Department of Psychiatry, The First Affiliated Hospital of Harbin Medical University, Harbin, Heilongjiang Province China; 3 School of Ethnology and Sociology, Yunnan University, Kunming, Yunnan Province China

**Keywords:** Depression, College students, Life satisfaction, Risky behavior

## Abstract

**Background:**

The Coronavirus disease 2019 pandemic spread rapidly worldwide, posing a serious health challenge to the global public. College students, lacking sufficient psychological resilience and coping skills, are more prone to depressive. Social isolation and online learning have led to increased health risk behaviors and decreased life satisfaction among college students. However, few studies exist on the association between health risk behaviors and depression among Chinese college students during the stage. Therefore, this study aims to investigate the prevalence of depression and its risk factors among college students during the pandemic.

**Methods:**

A total of 2150 first and second-year college students (whose age from 16 to 20) were recruited for this study from April to June 2020. Data were collected through the Adolescent Students’ Life Satisfaction Scale, Health Risk Behavior Scale, Self-rating Anxiety Scale, and Self-rating Depression Scale. A self-administered questionnaire collected other demographic data.Binary logistic regression was conducted to analyze the risk factors of depression.

**Results:**

The prevalence of depressive state of college students was 32.1%. Students with health-compromising behavior (OR = 1.128, *p* < 0.001) were positively correlated with depression. Furthermore, satisfaction with friendships (OR = 0.941, *p* < 0.001), freedom (OR = 0.955, *p* = 0.004), school (OR = 0.966, *p* = 0.010), and the environment (OR = 0.933, *p* < 0.001) were protective factors for students’ depression.

**Conclusions:**

To reduce this depression, schools should increase publicity and education to promote a regular diet among college students, channel adolescents’ destructive emotions to prevent them from self-injury and self-harm, enrich their after-school life to strengthen their interpersonal communication, and enhance friendship- building among them.

**Supplementary Information:**

The online version contains supplementary material available at 10.1186/s40359-025-03944-x.

## Background

Coronavirus disease 2019 (COVID-19) spread rapidly worldwide, posing a serious health challenge to the global public [1, 2]. Consequently, the public has experienced increased physical health problems and mental disorders such as anxiety, depression, and suicide [3, 4]. The impact of the pandemic serves as a greater challenge for adolescents because of their lack of mental capacity and coping skills compared to adults [5, 6]. Adolescents of varying backgrounds experienced higher rates of anxiety, depression, behavioral problems, and suicide during the pandemic. Globally, 10–20% of adolescents suffer from varying degrees of mental health [6, 7].

College marks an important stage for adolescents. Moreover, college students’ mental health has become an increasing concern, including issues related to panic, anxiety, and depression [8]. In general, incidences of depression among university students reached 20–50% during the COVID-19 pandemic [1, 9]. According to a large-scale survey of college students in China (*N* = 746,217), 21.1% of participants demonstrated symptoms of depression during the pandemic [10]. Many factors contributed to the high incidence of depression among college students during the COVID-19 pandemic, such as a constant fear of infection, social isolation, unhealthy lifestyles, and exposure to media information overload.

The impact of the COVID-19 pandemic on college students’ mental health is a global research focus. Due to differences in cultural, policy, and socioeconomic contexts across regions, risk factors share commonalities while also exhibiting distinct characteristics. A Chinese study identified fear of infection, academic pressure and challenges with online learning, social isolation, reduced physical activity and health status, family relationships, information overload, and rumors as primary risk factors [11]. Conversely, an American study found economic and employment stress, social disruption, disruption to daily routines, concerns about loved ones’ health, and housing-related stress to be key risk factors [12].

Notably, there was an upward trend in health risk behaviors among college students during the COVID-19 outbreak [11]. For example, they were restricted to online classes at home, which increased their health risk behaviors such as being sedentary and overeating. A lack of physical exercise and overeating is highly correlated with anxiety and depression symptoms and lower life satisfaction. In contrast, a healthy lifestyle that includes regular physical activity, a daily routine, good diet, and sufficient sleep help to decrease symptoms of depression and anxiety and improve life satisfaction.

Moreover, due to the lack of in-person support from teachers and interactive learning, online courses from home are likely to increase academic load and frustration, which may increase mental distress and decrease life satisfaction especially in terms of satisfaction with academics, school, and friendships [12]. College students with depression are more likely to cope with stress through negative coping strategies such as addiction behaviors and self-harm. Smartphone and Internet addiction are common among college students who resorted to connecting and socializing in this way because of orders to stay at home and school closures [13]. In addition, college students had a higher frequency of alcohol or cannabis use during the COVID-19 pandemic. Other coping strategies used by college students to deal with psychological problems during the COVID-19 pandemic included non-suicidal self-injury and suicide [14]. These negative coping skills increase the risk of depression among adolescents.

In China, the incidence of depression among college students totaled 20–50% during the COVID-19 pandemic [1, 9]. Unfortunately, few studies focus on the association between health risk behaviors, life Satisfaction and depression among Chinese college students. Furthermore, some studies indicate differences in health risk behaviors between Eastern and Western college students, including in weight perception, addiction behaviors, and the handling of stressful events [15]. Therefore, relevant studies in the West cannot be fully generalized to the situation in China. However, during the COVID-19 pandemic, the impact of the outbreak and the response policies varied significantly across different regions in China. Therefore, the primary objective of this study is to investigate the prevalence of depression among college students in Northeast China through a large-scale survey, examine the associations between depression, health risk behaviors, and life satisfaction, and analyze potential factors associated with depression.

## Methods

### Participants and procedures

A questionnaire survey was conducted from April to June 2020 among the first and second-year students of two randomly selected majors from two universities in Harbin, Heilongjiang Province in China. Before that, the investigators were trained by professionals to master the questionnaire method and its related notes (completeness of responses, information confidentiality, etc.). These aspects were explained to the students when they filled out the questionnaire. The investigators distributed the questionnaire to student administrators after communicating with each school’s student administration. The questionnaires were distributed to every class and retrieved immediately after students filled them out. In total, 2440 paper questionnaires were distributed, of which 2419 were retrieved. Among these, 2150 were valid after removing those with obvious errors, incorrect answers to lie detector questions, and incomplete answers, giving an effective response rate of 88.11%. All respondents signed an informed consent form.To address potential bias issues in the self-assessment questionnaire, we implemented the following measures: First, provided standardized instructions to ensure participants received consistent guidance before completing the questionnaire. Second, established a confidential environment enabling participants to complete the questionnaire in a relatively relaxed and secure state, while strictly safeguarding the confidentiality of responses. Third, maintained a neutral stance; all staff refrained from evaluating or discussing the content of returned questionnaires.Although cross-sectional designs have inherent limitations and cannot establish causation, they can still identify factors associated with depression among college students, providing valuable reference points for enhancing their mental health.

The inclusion criteria were as follows. Students had to (1) be aged 16–20 years, (2) be able to understand and complete the scale, and (3) voluntarily participate in the study. The exclusion criteria were as follows. Students who (1))History of psychotropic drug use or mental illness, and (2) did not have the ability to understand and fill in the questionnaire.

### Questionnaire

A self-designed demography questionnaire, the Life Satisfaction Scales Applicable to Chinese Adolescent Students, Adolescent Health Related Risky Behavior Inventory, Self-rating Anxiety Scale, and Self-rating Depression Scale were adopted in the study. Before the official survey, 100 college students were randomly selected from a university for the pre-survey. Based on the pre-survey results and expert opinions, the scale items were modified appropriately and the final questionnaire developed.

### Demographic characteristics

A self-designed demography questionnaire was used to collect data. Demographic information included 11 items pertaining to gender, age, grade, place of residence, being an only child or not, monthly family income, parents’ educational background, parents’ employment, and being physically punished by parents in childhood.

### Life satisfaction scales applicable to Chinese adolescent students

The Life Satisfaction Scales Applicable to Chinese Adolescent Students (CASLSS), developed by Zhang et al. [16], is based on the Adolescent Multidimensional Life Satisfaction Scale by Huebener [17]. CASLSS includes six dimensions: friendship, family, study, freedom, school, and environment. The scale includes 36 items scored on a seven-point Likert scale ranging from “1 = Completely inconsistent” to “7 = Completely consistent”. The higher the score, the higher the satisfaction. In this study, the Cronbach’s coefficients of each dimension were 0.71, 085, 0.79, 0.87, and 0.71, respectively, and the Cronbach’s coefficient of the entire scale was 0.91.

### Adolescent health related risky behavior inventory

The Adolescent Health Related Risky Behavior Inventory (AHRBI) by Wang Mengcheng [18], a Chinese scholar, and others includes six dimensions: health compromise, violent aggression, suicide and self-harm, smoking and drinking, discipline violation, and unprotected sex. It includes 38 items scored on a five-point Likert scale (0 = never, 1 = almost never (once a month), 2 = sometimes (2–4 times a month), 3 = almost often (2–3 times a week), 4 = often (more than 4 times a week)). The detection rate for each dimension was calculated based on at least one of the items included in the dimension ≥ 2 points (2–4 times a month). Cronbach’s coefficient for all scales was 0.90, and that of each scale was mostly above 0.75.

### Self-rating anxiety scale

The Self-rating Anxiety Scale (SAS) by Zung [19]employs a four-point Likert scale, i.e., the scores of 20 items added together are the total gross score, and the integer from the total gross score times 1.25 is the standard score. A standard score of < 50 indicates no anxiety, 50–59 indicates mild anxiety, 60–69 indicates moderate anxiety, and ≥ 70 indicates severe anxiety. Cronbach’s coefficient was 0.697. In this study, the Cronbach’s coefficient was 0.697. It is barely acceptable.This may be attributed to the narrow range of trait distribution within the sample or the need for further validation of the scale’s applicability within this cultural context.

### Self-rating depression scale

The Self-rating Depression Scale (SDS), also by Zung [20], comprises 20 items.The scores of 20 items added together are the total gross score, and the integer from the total gross score times 1.25 is the standard score. A standard score of < 50 indicates no depression, 50–59 indicates mild depression, 60–69 indicates moderate depression, and ≥ 70 indicates severe depression. Cronbach’s coefficient was 0.86.

### Data analysis

All patients’demographic variables were tested for normal distribution (Kolmogorov-Smirnov one-sample test, all *p* > 0.05); all data were compared between patients with and without depression. We used analysis of variance (ANOVA) for continuous variables an chi-square tests for categorical variables. The prevalence of depression was analyzed by the chi-square test. We used the Bonferroni corrections to adjust for multiple testing. We diagnose the collinearity of significant variables and manually delete the variables with collinearity. Finally, the stepwise logistic regression analysis (forward selection) was used to assess correlations between depression and the variables that were passed the Bonferroni corrections in Table 2. We used IBM SPSS26.0 to perform all statistical analyses. All *p* values were calculated with a 2-tailed significance level of < 0.05.

## Results

### Demographic characteristics of the respondents

The college students participating in the survey were mainly male (54.8%), aged 19 years (44.0%), freshmen (59%), urban (63.7%), only children (64.1%), and from families earning 4001–8000 RMB family income per month (39.7%). Table 1 provides more details.

**Table 1:** Demographic characteristics of participants (*N* = 2150)

Variables	*N*	%
Gender		
Male	1 179	54.8
Female	971	45.2
Age group		
16–18 years old	677	31.5
19 years old	946	44.0
20 years old	527	24.5
Grade		
Freshman	1 268	59.0
Sophomore	882	41.0
Place of family residence		
Country	781	36.3
Urban	1 369	63.7
Only child or not		
Only children	1 379	64.1
Non- only children	771	35.9
Family income per month		
<4 000 yuan	607	28.2
4 001–8 000 yuan	853	39.7
>8 001yuan	690	32.1
Father’s educational level		
Mainly junior high school/primary school or less	741	34.5
High school or technical secondary school	689	32.0
College or above	720	33.5
Father’s occupation		
Individual, business and service staff	600	27.9
Farmer	368	17.1
Professionals	225	10.5
Personnel of government organs and public institutions	454	21.1
Staff of the enterprise	213	9.9
Other	290	13.5
Mother’s educational level		
Mainly junior high school, primary school or less	886	41.2
High school or technical secondary school	686	31.9
College or above	578	26.9
Mother’s occupation		
Individual, business and service staff	604	28.0
Farmer	380	17.7
Professionals	139	6.5
Personnel of government organs and public institutions	425	19.8
Staff of the enterprise	182	8.5
Other	420	19.5
Physically punished in childhood		
No	895	41.6
Yes	1255	58.4

### Differences in depression scores of college students for diverse variables

The results showed that the prevalence of depressive state of college students is 32.1% (692/2150). Compared with the non-depression group, the monthly family income (x^2^= 16.437, *p* < 0.001), and physical punishment in childhood (x^2^ = 13.383, *p* < 0.001), however the depressive state of the depression group demonstrated significant differences in family residence (x^2^ = 4.309, *p* = 0.039), father’s educational level (x^2^ = 7.907, *p* = 0.019), mother’s educational level (x^2^ = 6.217, *p* = 0.045) did not pass the Bonferroni corrections (Bonferroni corrected *p* < 0.05/20 = 0.0025). Further details are provided in Table 2. 

**Table 2:** Differences in depression scores of college students for diverse variables

Variables	with depression	without depression	F/x^2^	*p*
Age	18.955 ± 0.897	18.951 ± 0.874	0.009	0.924
Gender (female)	297(42.9%)	674(46.2%)	2.074	0.151
Place of family residence (country)	273(39.5%)	508(34.8%)	4.309	0.039
only children	441(63.7%)	938(64.3%)	0.075	0.810
Family income per month			16.437	< 0.001
Less 4000(yuan)	226(32.7%)	381(26.1%)		
4000–8000(yuan)	281(40.6%)	572(39.2%)		
Over 8000(yuan)	185(26.7%)	505(34.6%)		
Father’s occupation			7.907	0.019
Junior or primary school	254(36.7%)	487(33.4%)		
High or technical secondary school	235(34.0%)	454(31.1%)		
College or above	203(29.3%)	517(35.5%)		
Mother’s educational level			6.217	0.045
Junior or primary school	282(40.8%)	604(41.4%)		
High or technical secondary school	243(35.1%)	443(30.4%)		
College or above	167(24.1%)	411(28.2%)		
Physically punished(yes)	443(64.0%)	812(55.7%)	13.383	< 0.001
Health Compromise	3.684 ± 3.235	1.877 ± 1.971	255.256	< 0.001
Violent attacks	4.390 ± 5.796	1.881 ± 2.044	216.628	< 0.001
Suicide and self-mutilation	1.734 ± 3.010	0.207 ± 0.743	332.783	< 0.001
Smoking and drinking	2.270 ± 4.151	0.644 ± 1.470	177.136	< 0.001
Breaking the rules	3.046 ± 4.121	1.381 ± 1.476	187.389	< 0.001
Unprotected	0.993 ± 2.965	0.049 ± 0.598	136.020	< 0.001
Friendship satisfaction	32.921 ± 7.746	39.602 ± 5.492	526.594	< 0.001
Family satisfaction	34.538 ± 9.206	41.797 ± 6.628	433.439	< 0.001
School satisfaction	26.383 ± 6.518	32.746 ± 6.300	468.134	< 0.001
Academic satisfaction	23.393 ± 7.634	28.453 ± 6.893	235.660	< 0.001
Freedom satisfaction	23.145 ± 5.995	28.474 ± 4.720	499.763	< 0.001
Environmental satisfaction	22.439 ± 4.840	27.466 ± 4.900	497.871	< 0.001

### Results of binary logic regression analysis

The results in Table 3 show that health-compromising behavior (odd ratio = 1.128, 95% CI = 1.017–1.188, Wald X^2^ = 20.826, *p* < 0.001), suicide and self-harm(odd ratio = 1.470, 95% CI = 1.309–1.651, Wald X^2^ = 42.123, *p* < 0.001), friendship satisfaction(odd ratio = 0.941, 95% CI = 0.915–0.969, Wald X^2^ = 17.091, *p* < 0.001), freedom satisfaction(odd ratio = 0.955, 95% CI = 0.926–0.985, Wald X^2^ = 8.516, *p* = 0.004), school satisfaction(odd ratio = 0.966, 95% CI = 0.941–0.992, Wald X^2^ = 6.560, *p* = 0.01), and environment satisfaction(odd ratio = 0.933, 95% CI = 0.903–0.965, Wald X^2^ = 16.885, *p* < 0.001) are related factors for college students’ depressive state. Furthermore, college students with health-compromising behavior had a clear positive correlation with a depressive state, that is, college students with health compromising behavior were at a higher risk of depression. Furthermore, college students satisfied with school had lower depression than those dissatisfied with it. Finally, students satisfied with the environment had lower depression than dissatisfied with it (Table 3).We found a statistically significant association between these factors and depression. However, the OR was close to 1, indicating an extremely small effect, despite its 95%CI not containing 1. Although statistically significant in a large sample, this association may lack clinical or practical significance and is provided for reference by relevant institutions only.

**Table 3:** Binary logistic regression analysis

Variables	B	Wsld X^2^	OR	95% CI	*P*
Total score of Health Compromise	0.121	20.826	1.128	1.017–1.188	<0.001
Total score of suicide and self-mutilation	0.385	42.123	1.470	1.309–1.651	<0.001
Friendship satisfaction	−0.060	17.091	0.941	0.915–0.969	<0.001
Freedom satisfaction	−0.046	8.516	0.955	0.926–0.985	0.004
School satisfaction	−0.034	6.560	0.966	0.941–0.992	0.010
Environmental satisfaction	−0.069	16.885	0.933	0.903–0.965	<0.001

## Discussion

This study was a large sample epidemiological survey of university students in northeastern China. The study investigated the main risk factors for a depressive state among college students in northeastern China. Our findings revealed significant differences between college students with and without depression in terms of place of residence, family income, physical punishment during childhood, adolescent health-related risk behaviors, and adolescent life satisfaction. Further exploration revealed that health-compromising behavior, suicidal and self-injury behaviors, school satisfaction, friendship satisfaction, freedom satisfaction, and environmental satisfaction were related factors for a depressed state among college students.

Our study found that the higher the health-compromising behavior score of college students, the more severe was their depression. Health-related risk behaviors included aggressive violence, disciplinary disruption, suicidal self-injury, health-compromising behaviors, unprotected sexual behaviors, smoking, and drinking [21]. In this study, we found that college students with depression had higher total health-compromising behavior scores, higher total scores for violent aggression, more suicidal self-injurious behaviors, were more likely to break discipline, and smoked and drank alcohol more. Further study found that health-compromising behavior is a related factor for a depressed state among college students. An unhealthy diet and lack of physical activity also negatively affect health and are therefore considered health risk behaviors [22]. Health promotion and incorporating health-protective behaviors into people’s lifestyles have gone a long way in recent years to improve individuals’ overall health and well-being. Studies have found that college students are at increased risk of developing unhealthy diets and lifestyles [23]. Several studies supported our results that a lack of physical activity increases health risk [24]. Prolonged sitting can increase the risk of developing chronic conditions like obesity, metabolic syndrome, and depression [25]. In addition, as shown in the literature, skipping breakfast is common among college students. Mansouri et al. [26] reported that regular breakfast consumption was negatively associated with overweight and obesity among college students. Poor eating behaviors among college students may increase the risk of developing mood disorders, and understanding their overall eating behaviors may be necessary to reduce this risk. A logical explanation may be that when college students with depression fail to follow general health advice pertaining to moderate eating or eating breakfast on time, they may not develop good eating habits, but instead overeat, diet excessively, or skip breakfast. A comparative study showed that students who perceived their health as good, very good, or excellent had healthier behaviors, and that university health and wellness initiatives should focus on the mental and physical health of college students to help them develop positive behaviors that affect overall health-related quality of life. Therefore, health-related risk behaviors should be a point of focus, as these will likely persist into adult life with various negative consequences.

Our findings showed that suicidal and self-injury behaviors were positively associated with a depressed state among college students. Previous studies have related depression with the severity of suicidal ideation in a sample of college students at high risk of suicidal behavior due to past suicide attempts [27]. This finding is consistent with the results of our study. Suicidal behavior among college students is associated with various factors such as psychopathology, stressful life events, personality traits, the rapid growth of higher education, and high expectations students must meet, all of which increase their psychological stress. Anxiety and despair due to the aforementioned factors can moderate the relationship between stress and depressive symptoms and promote the onset of depression [28]. The occurrence of suicidal behavior and depression may also be related to school belonging. A recent study showed that the interaction between loneliness and school belonging increased the risk of suicide and the resulting risk such as suicidal behavior and depression, which could serve as predictors [29]. These findings suggest that suicidal behavior and the occurrence of depression are closely related. Non-suicidal self-injury (NSSI) behavior among college students is a current social concern. One study showed that NSSI behavior has specific emotional characteristics such as eating disorders, depression, anxiety, and stress. Moreover, college students with prior experience of NSSI have fewer adaptive coping strategies when they encounter problems that may lead to emotional difficulties such as the emergence of a depressed mood, which supports our findings. The association between NSSI and depression may be closely related to neuroticism. Studies have shown that neuroticism significantly negatively predicts mood regulation, and positively predicts depression and NSSI [30]. Highly neurotic people are prone to negative emotions such as anger, shame, and guilt. When unable to deal with these emotions effectively, they seek mental stimulation by punishing themselves or through NSSI. Other negative emotions, especially depression, are also generated. NSSI behaviors and a depressed state in patients with high neuroticism are closely related [31]. Notably, previous studies have linked internet addiction and media exposure to increased negative emotions among students. During the COVID-19 pandemic, students are more likely to be exposed to the internet and be more easily distracted by the mixed bag of negative information it contains. This may prompt them to shift from self-harming thoughts to self-harming behaviors and exacerbate depression.Our findings suggest that we should be highly concerned about the possibility of depression among college students with a history of suicidal or self-injurious behavior.

Life satisfaction is a cognitive component of an individual’s subjective well-being, and is closely related to the psychological health of college students [32]. Survey results regarding college students’ life satisfaction significantly related to their social, behavioral, and psychological problems (depression and anxiety). The results of this study showed that the life satisfaction of college students with depression was significantly different from that of those who were not depressed in all six dimensions (friendship, family, school, academics, freedom, and environment) (Table 1, all *p* < 0.001) [33]. Consistent with the results of this study, a Chinese study identified fear of infection, academic pressure and challenges with online learning, social isolation, reduced physical activity and health status, family relationships, information overload, and rumors as primary risk factors [11]. Conversely, an American study found economic and employment stress, social disruption, disruption to daily routines, concerns about loved ones’ health, and housing-related stress to be key risk factor [12].The likely reason lies in resistance to restrictions on personal freedom: lockdown and social distancing measures clash sharply with the emphasis on liberty and autonomy within individualistic cultures. Consequently, the disruption of daily routines and social isolation may provoke more intense feelings of frustration and psychological stress among American students [11].In addition, many Chinese college students returned to live with their families during the pandemic. Consequently, the quality of family relationships emerged as a decisive factor. Harmonious families served as a powerful protective factor, while family conflicts became a significant risk factor. At the same time, concerns about the health of vulnerable family members, such as grandparents, became particularly prominent [12]. In the West, Many students live away from home on campus, and even during lockdowns, they may choose to live alone or with roommates rather than return to their family homes. Consequently, the social support they perceive comes more from peers and romantic relationships. When these relationships are disrupted by isolation, they experience deeper loneliness. While family support remains important, physical distance limits its effectiveness [34].

A Chinese questionnaire survey of 439 college students aged 17–24 years found that life satisfaction was positively associated with self-esteem and social support and negatively associated with variables such as depression and suicidal ideation [35]. Similarly, a US study of 508 full-time undergraduate students aged 18–24 years found that life satisfaction was a significant independent predictor of depression. In addition, a study investigating 1,224 university students in Brazil during the COVID-19 pandemic found that depression and anxiety were negatively associated with life satisfaction, mental health, and adaptive coping styles, and positively associated with poor coping styles. Many results support a negative correlation between life satisfaction and depression [36, 37]. To explain this phenomenon, some scholars have proposed that the generation of depression and anxiety may be related to people’s inability to satisfy their needs in valuable areas of their lives. As low satisfaction does not bring people happy emotions, this leads to depression [38]. In conclusion, effectively improving college students’ life satisfaction is beneficial to their mental health and may help reduce their risk of depression. It is noteworthy that although an increasing number of studies link mental health and life satisfaction, few have refined the different dimensions of life satisfaction among college students.

A logistic regression in this study revealed that school satisfaction, friendship satisfaction, freedom satisfaction, and environmental satisfaction were related factors for a depressed state among college students. Universities are advised to foster a free and equal atmosphere, improve the learning and living environment, actively conduct satisfaction surveys, and, when necessary, invite professional psychiatrists to provide targeted interpersonal psychotherapy to enhance students’ psychological ownership, which is crucial for boosting vitality and alleviating depression [38]. It is recommended that teachers try to become friends with students and adopt methods such as positive gossip to buffer students’ negative psychological conditions and reduce psychological alienation [39].Students are advised to set and actively pursue periodic goals, leverage their strengths, and address their weaknesses, thereby increasing their life satisfaction and demonstrating a higher level of self-efficacy [40].

Note that this study did not find that family and academic satisfaction significantly predicted a depressed state among college students. This differs from the results of previous studies [41], which may be related to cultural differences. China tends to have a strict entrance scoreline for college students, but it is relatively easy to study after enrollment. However, in some Western countries, the opposite is true [40]. In addition, most of the residence patterns of Chinese university students are residential, and few students live with their families. When faced with more challenges and tasks, the emotional impact of family on them may be diluted. In conclusion, it is recommended that university health professionals have a targeted focus on college students’ life satisfaction and implement strategies and programs to reduce their depression.

### Limitations of this study

This study has some limitations. First, it is cross-sectional and cannot infer an actual causal relationship. In the future, we could conduct a longitudinal follow-up study to clarify the results. Specifically, a prospective cohort can be established when freshmen enter college and followed up multiple times throughout their college years (or even after graduation). This design can not only reveal the causal temporal relationship between risky health behaviors, life satisfaction, and depressive symptoms, but also identify dynamic predictors of depression trajectories, such as which factors make students show psychological resilience or susceptibility under epidemic stress or normal stress. Second, The data in this study relied primarily on self-reports and may therefore be affected by factors such as social desirability bias and recall bias. Although we mitigated these issues by ensuring anonymity, using standardized instruments, and statistically verifying the low level of common method bias (using Harman’s single-factor test), future studies could employ methods such as behavioral observation and clinical interviews to provide more comprehensive evidence. Third, Although we examined a range of risk factors, several important potential confounders, such as substance use, physical health indicators, and genetic factors, were not included. Future studies should integrate more comprehensive biopsychosocial indicators, such as body mass index and medical history obtained through medical records, and genotyping through biospecimen collection to construct more complex, multilevel prediction models of depression risk. Fourth, in this study, the Cronbach’s α coefficient for the Self-Rating Anxiety Scale was 0.697, slightly lower than the standard of 0.70. This may be due to the specificity or sample size of this study. Future research could consider validating the reliability and validity of the scale within the specific population targeted by this study or developing and implementing more culturally sensitive and population-specific assessment tools. Fifth, the sample size of this study was selected from two universities in Northeast China, which limits the generalizability of the results. To enhance the external validity and robustness of the findings, future research should be conducted through nationwide, multicenter collaborations. A stratified random sampling approach is recommended, drawing representative samples from different geographic regions (eastern, western, southern, northern, and central China) and different types of universities (e.g., comprehensive universities, polytechnics, and vocational colleges). This design would effectively test whether the risk factors identified in this study in Northeast China hold true across diverse cultural and educational contexts.Thus, the findings of this study cannot be extended to all Chinese college students. Finally, This study focused on prevalence and related factors, lacking a clear healthy control group for in-depth intergroup comparisons. Future research could, based on longitudinal cohort data, clearly define a “depressed group” and a “healthy control group” based on depression trajectories. Using case-control studies, these studies could further compare the fundamental differences between the two groups in terms of genetics, psychological traits, social support networks, and health behaviors, thereby providing targets for targeted psychological interventions.

## Conclusions

During the COVID-19 pandemic, college students experienced a rise in depression rates due to multiple factors. To reduce the incidence of depression, universities should strengthen educational outreach emphasizing the importance of regular eating habits, consistent sleep schedules, and healthy lifestyles. Timely intervention is crucial for alleviating negative emotions among young people and preventing self-harm behaviors. Additionally, universities should offer more diverse extracurricular activities to enhance students’ interpersonal skills, foster friendships among peers, and thereby increase their overall life satisfaction and friendship fulfillment. Overall, reducing harmful behaviors among youth and improving their life satisfaction are essential for lowering depression rates among young adults.This experience can also be applied to other emergency situations. 

## Supplementary Information

Supplementary Material 1.

Supplementary Material 2.

Supplementary Material 3.

Supplementary Material 4.

Supplementary Material 5.

## Data Availability

The data that support the findings of this study are available from the corresponding author N.Z. upon reasonable request.

## Abbreviations

COVID-19: Coronavirus disease 2019
CASLSS: Life Satisfaction Scales Applicable to Chinese Adolescent Students
AHRBI: Adolescent Health Related Risky Behavior Inventory
SAS: Self-rating Anxiety Scale
SDS: Self-rating Depression Scale
