# Supplementary material for: Prevalence and risk factors of depression in college students in Northeast China during the COVID-19 pandemic: a cross-sectional study
Source: BMC Psychol. 2026 Jan 7;14:171. doi: 10.1186/s40359-025-03944-x (PMC12869974; doi:10.1186/s40359-025-03944-x)
Supplement: Supplementary file 1 — Supplementary Material 1. [file 40359_2025_3944_MOESM1_ESM.docx]

****Self-Rating Anxiety Scale (SAS)****

****Instructions:**** Below is a list of 20 statements. Please read each one carefully and indicate how frequently you have experienced each symptom ****over the past week**** by selecting the most appropriate option.

| **No.** | **Question** | **A little or none of the time** | **Some of the time** | **A good part of the time** | **Most or all of the time** |
| --- | --- | --- | --- | --- | --- |
| 1 | I feel more nervous and anxious than usual | 1 | 2 | 3 | 4 |
| 2 | I feel afraid for no reason | 1 | 2 | 3 | 4 |
| 3 | I get upset easily or feel panicky | 1 | 2 | 3 | 4 |
| 4 | I feel like I'm going to fall apart | 1 | 2 | 3 | 4 |
| 5 | I feel that everything is fine and nothing bad will happen | 1 | 2 | 3 | 4 |
| 6 | My arms and legs shake and tremble | 1 | 2 | 3 | 4 |
| 7 | I am bothered by headaches, neck and back pain | 1 | 2 | 3 | 4 |
| 8 | I feel weak and get tired easily | 1 | 2 | 3 | 4 |
| 9 | I feel calm and can sit still easily | 1 | 2 | 3 | 4 |
| 10 | I can feel my heart beating fast | 1 | 2 | 3 | 4 |
| 11 | I am bothered by dizzy spells | 1 | 2 | 3 | 4 |
| 12 | I have fainting feelings or feel faint | 1 | 2 | 3 | 4 |
| 13 | I can breathe in and out easily | 1 | 2 | 3 | 4 |
| 14 | I get feelings of numbness and tingling in my fingers and toes | 1 | 2 | 3 | 4 |
| 15 | I am bothered by stomach aches or indigestion | 1 | 2 | 3 | 4 |
| 16 | I have to empty my bladder often | 1 | 2 | 3 | 4 |
| 17 | My hands are usually dry and warm | 1 | 2 | 3 | 4 |
| 18 | My face gets hot and blushes | 1 | 2 | 3 | 4 |
| 19 | I fall asleep easily and get a good night's sleep | 1 | 2 | 3 | 4 |
| 20 | I have nightmares | 1 | 2 | 3 | 4 |
