# Supplementary material for: Prevalence and risk factors of depression in college students in Northeast China during the COVID-19 pandemic: a cross-sectional study
Source: BMC Psychol. 2026 Jan 7;14:171. doi: 10.1186/s40359-025-03944-x (PMC12869974; doi:10.1186/s40359-025-03944-x)
Supplement: Supplementary file 2 — Supplementary Material 2. [file 40359_2025_3944_MOESM2_ESM.docx]

****Adolescent Life Satisfaction Scale****

****Dimension Mapping:Friendship Satisfaction:**** Items 1, 7, 13, 19, 25, 31, 35

****Family Satisfaction:**** Items 2, 8, 14, 20, 26, 32, 36

****School Satisfaction:**** Items 3, 9, 15, 21, 27, 33

****Academic Satisfaction:**** Items 6, 12, 18, 24, 30, 34

****Freedom Satisfaction:**** Items 5, 11, 17, 23, 29

****Environment Satisfaction:**** Items 4, 10, 16, 22, 28

Note: (R) indicates reverse-scored items.

****Instructions:**** Please read each statement and indicate how much you agree or disagree by circling the number that best matches your feeling.

| **Item** | **Statement** | **Response Scale** |  |  |  |  |  |  |
| --- | --- | --- | --- | --- | --- | --- | --- | --- |
| 1 | My friends respect me. | 1 - Strongly Disagree | 2 - Disagree | 3 - Slightly Disagree | 4 - Neutral | 5 - Slightly Agree | 6 - Agree | 7 - Strongly Agree |
| 2 | I enjoy spending time with my parents. | 1 - Strongly Disagree | 2 - Disagree | 3 - Slightly Disagree | 4 - Neutral | 5 - Slightly Agree | 6 - Agree | 7 - Strongly Agree |
| 3 | I feel uncomfortable at school. (R) | 1 - Strongly Disagree | 2 - Disagree | 3 - Slightly Disagree | 4 - Neutral | 5 - Slightly Agree | 6 - Agree | 7 - Strongly Agree |
| 4 | I wish I lived somewhere else, not where I live now. (R) | 1 - Strongly Disagree | 2 - Disagree | 3 - Slightly Disagree | 4 - Neutral | 5 - Slightly Agree | 6 - Agree | 7 - Strongly Agree |
| 5 | Basically, no one forces me to do things I don't like. | 1 - Strongly Disagree | 2 - Disagree | 3 - Slightly Disagree | 4 - Neutral | 5 - Slightly Agree | 6 - Agree | 7 - Strongly Agree |
| 6 | I have achieved my ideal accomplishments in my studies. | 1 - Strongly Disagree | 2 - Disagree | 3 - Slightly Disagree | 4 - Neutral | 5 - Slightly Agree | 6 - Agree | 7 - Strongly Agree |
| 7 | I have many friends. | 1 - Strongly Disagree | 2 - Disagree | 3 - Slightly Disagree | 4 - Neutral | 5 - Slightly Agree | 6 - Agree | 7 - Strongly Agree |
| 8 | My family is a happy family. | 1 - Strongly Disagree | 2 - Disagree | 3 - Slightly Disagree | 4 - Neutral | 5 - Slightly Agree | 6 - Agree | 7 - Strongly Agree |
| 9 | There are many things about school that I don't like. (R) | 1 - Strongly Disagree | 2 - Disagree | 3 - Slightly Disagree | 4 - Neutral | 5 - Slightly Agree | 6 - Agree | 7 - Strongly Agree |
| 10 | There are many unsatisfactory things in the environment where I live. (R) | 1 - Strongly Disagree | 2 - Disagree | 3 - Slightly Disagree | 4 - Neutral | 5 - Slightly Agree | 6 - Agree | 7 - Strongly Agree |
| 11 | I can generally act according to my own wishes. | 1 - Strongly Disagree | 2 - Disagree | 3 - Slightly Disagree | 4 - Neutral | 5 - Slightly Agree | 6 - Agree | 7 - Strongly Agree |
| 12 | I am satisfied with my academic situation. | 1 - Strongly Disagree | 2 - Disagree | 3 - Slightly Disagree | 4 - Neutral | 5 - Slightly Agree | 6 - Agree | 7 - Strongly Agree |
| 13 | My friends would help me if I needed it. | 1 - Strongly Disagree | 2 - Disagree | 3 - Slightly Disagree | 4 - Neutral | 5 - Slightly Agree | 6 - Agree | 7 - Strongly Agree |
| 14 | Most of the time, I like my parents' way of educating me. | 1 - Strongly Disagree | 2 - Disagree | 3 - Slightly Disagree | 4 - Neutral | 5 - Slightly Agree | 6 - Agree | 7 - Strongly Agree |
| 15 | I like going to school. | 1 - Strongly Disagree | 2 - Disagree | 3 - Slightly Disagree | 4 - Neutral | 5 - Slightly Agree | 6 - Agree | 7 - Strongly Agree |
| 16 | The public security is good where I live. | 1 - Strongly Disagree | 2 - Disagree | 3 - Slightly Disagree | 4 - Neutral | 5 - Slightly Agree | 6 - Agree | 7 - Strongly Agree |
| 17 | I basically have the freedom to make my own choices. | 1 - Strongly Disagree | 2 - Disagree | 3 - Slightly Disagree | 4 - Neutral | 5 - Slightly Agree | 6 - Agree | 7 - Strongly Agree |
| 18 | Compared to most of my classmates, my overall development at school is more comprehensive. | 1 - Strongly Disagree | 2 - Disagree | 3 - Slightly Disagree | 4 - Neutral | 5 - Slightly Agree | 6 - Agree | 7 - Strongly Agree |
| 19 | My friends treat me well. | 1 - Strongly Disagree | 2 - Disagree | 3 - Slightly Disagree | 4 - Neutral | 5 - Slightly Agree | 6 - Agree | 7 - Strongly Agree |
| 20 | My family members get along harmoniously with each other. | 1 - Strongly Disagree | 2 - Disagree | 3 - Slightly Disagree | 4 - Neutral | 5 - Slightly Agree | 6 - Agree | 7 - Strongly Agree |
| 21 | I like school life. | 1 - Strongly Disagree | 2 - Disagree | 3 - Slightly Disagree | 4 - Neutral | 5 - Slightly Agree | 6 - Agree | 7 - Strongly Agree |
| 22 | The social atmosphere is good where I live. | 1 - Strongly Disagree | 2 - Disagree | 3 - Slightly Disagree | 4 - Neutral | 5 - Slightly Agree | 6 - Agree | 7 - Strongly Agree |
| 23 | I can do things I like in my spare time. | 1 - Strongly Disagree | 2 - Disagree | 3 - Slightly Disagree | 4 - Neutral | 5 - Slightly Agree | 6 - Agree | 7 - Strongly Agree |
| 24 | Compared to my classmates, I have received more honors/awards at school. | 1 - Strongly Disagree | 2 - Disagree | 3 - Slightly Disagree | 4 - Neutral | 5 - Slightly Agree | 6 - Agree | 7 - Strongly Agree |
| 25 | I have high prestige among my peers. | 1 - Strongly Disagree | 2 - Disagree | 3 - Slightly Disagree | 4 - Neutral | 5 - Slightly Agree | 6 - Agree | 7 - Strongly Agree |
| 26 | My parents treat me as an equal. | 1 - Strongly Disagree | 2 - Disagree | 3 - Slightly Disagree | 4 - Neutral | 5 - Slightly Agree | 6 - Agree | 7 - Strongly Agree |
| 27 | I like school activities. | 1 - Strongly Disagree | 2 - Disagree | 3 - Slightly Disagree | 4 - Neutral | 5 - Slightly Agree | 6 - Agree | 7 - Strongly Agree |
| 28 | The world we live in is peaceful and tranquil. | 1 - Strongly Disagree | 2 - Disagree | 3 - Slightly Disagree | 4 - Neutral | 5 - Slightly Agree | 6 - Agree | 7 - Strongly Agree |
| 29 | Basically, no one interferes with my life. | 1 - Strongly Disagree | 2 - Disagree | 3 - Slightly Disagree | 4 - Neutral | 5 - Slightly Agree | 6 - Agree | 7 - Strongly Agree |
| 30 | I feel I have high status/face among my peers. | 1 - Strongly Disagree | 2 - Disagree | 3 - Slightly Disagree | 4 - Neutral | 5 - Slightly Agree | 6 - Agree | 7 - Strongly Agree |
| 31 | I wish I had different friends than the ones I have now. (R) | 1 - Strongly Disagree | 2 - Disagree | 3 - Slightly Disagree | 4 - Neutral | 5 - Slightly Agree | 6 - Agree | 7 - Strongly Agree |
| 32 | My family members speak to each other in a friendly manner. | 1 - Strongly Disagree | 2 - Disagree | 3 - Slightly Disagree | 4 - Neutral | 5 - Slightly Agree | 6 - Agree | 7 - Strongly Agree |
| 33 | My life at school is interesting. | 1 - Strongly Disagree | 2 - Disagree | 3 - Slightly Disagree | 4 - Neutral | 5 - Slightly Agree | 6 - Agree | 7 - Strongly Agree |
| 34 | I have a strong sense of achievement in my studies. | 1 - Strongly Disagree | 2 - Disagree | 3 - Slightly Disagree | 4 - Neutral | 5 - Slightly Agree | 6 - Agree | 7 - Strongly Agree |
| 35 | I have a lot of fun with my friends. | 1 - Strongly Disagree | 2 - Disagree | 3 - Slightly Disagree | 4 - Neutral | 5 - Slightly Agree | 6 - Agree | 7 - Strongly Agree |
| 36 | I can talk happily with my parents. | 1 - Strongly Disagree | 2 - Disagree | 3 - Slightly Disagree | 4 - Neutral | 5 - Slightly Agree | 6 - Agree | 7 - Strongly Agree |
