# Supplementary material for: Prevalence and risk factors of depression in college students in Northeast China during the COVID-19 pandemic: a cross-sectional study
Source: BMC Psychol. 2026 Jan 7;14:171. doi: 10.1186/s40359-025-03944-x (PMC12869974; doi:10.1186/s40359-025-03944-x)
Supplement: Supplementary file 3 — Supplementary Material 3. [file 40359_2025_3944_MOESM3_ESM.docx]

****Adolescent Risk Behavior Scale****

****Scale Dimensions:****

Health Compromising Behaviors (Items 1, 4, 19, 24, 31)

Smoking & Drinking (Items 6, 9, 10, 14, 17, 22)

Discipline Violation (Items 8, 11, 18, 20, 23, 27, 28)

Suicide & Self-harm (Items 5, 12, 21, 25, 36)

Violent & Aggressive Behavior (Items 2, 3, 7, 13, 15, 26, 29, 30, 33, 37)

Unprotected Sexual Behavior (Items 16, 32, 34, 35, 38)

| **No.** | **Question** | **Never** | **Rarely (About once a month)** | **Sometimes (2-4 times a month)** | **Often (2-3 times a week)** | **Very Often (4+ times a week)** |
| --- | --- | --- | --- | --- | --- | --- |
| 1 | Do you eat breakfast? | 0 | 1 | 2 | 3 | 4 |
| 2 | Have you verbally attacked others? | 0 | 1 | 2 | 3 | 4 |
| 3 | Have you damaged property (not your own)? | 0 | 1 | 2 | 3 | 4 |
| 4 | Do you drink milk or soy milk? | 0 | 1 | 2 | 3 | 4 |
| 5 | Have you ever tried to cut or burn yourself? | 0 | 1 | 2 | 3 | 4 |
| 6 | Do you smoke? | 0 | 1 | 2 | 3 | 4 |
| 7 | Have you ever maliciously teased someone because of their physical disability or appearance? | 0 | 1 | 2 | 3 | 4 |
| 8 | Have you ever skipped classes or played truant for a whole day? | 0 | 1 | 2 | 3 | 4 |
| 9 | Have you ever smoked because peers persuaded you to? | 0 | 1 | 2 | 3 | 4 |
| 10 | Have you ever binge drank or gotten drunk? | 0 | 1 | 2 | 3 | 4 |
| 11 | Have you ever run away from home? | 0 | 1 | 2 | 3 | 4 |
| 12 | Have you had thoughts of suicide? | 0 | 1 | 2 | 3 | 4 |
| 13 | Have you ever bullied, threatened, or intimidated your peers? | 0 | 1 | 2 | 3 | 4 |
| 14 | Do you feel irritable, lose your temper, or experience headaches/insomnia when you don't have cigarettes to smoke? | 0 | 1 | 2 | 3 | 4 |
| 15 | Have you been involved in a physical fight? | 0 | 1 | 2 | 3 | 4 |
| 16 | Have you engaged in unsafe sexual behavior? | 0 | 1 | 2 | 3 | 4 |
| 17 | At classmate gatherings, do you drink alcohol excessively? | 0 | 1 | 2 | 3 | 4 |
| 18 | Have you ever received a warning, demerit, disciplinary action, or been expelled from school for violating rules? | 0 | 1 | 2 | 3 | 4 |
| 19 | Do you not participate in any form of physical activity? | 0 | 1 | 2 | 3 | 4 |
| 20 | Have you ever cheated or plagiarized? | 0 | 1 | 2 | 3 | 4 |
| 21 | Have you ever intentionally injured yourself by biting, scratching, hitting yourself, etc.? | 0 | 1 | 2 | 3 | 4 |
| 22 | Do you feel it's losing face if you don't drink when others are drinking? | 0 | 1 | 2 | 3 | 4 |
| 23 | Have you ever lied to your family members (e.g., grandparents, parents, siblings)? | 0 | 1 | 2 | 3 | 4 |
| 24 | Have you engaged in binge eating or purging behavior (vomiting after eating)? | 0 | 1 | 2 | 3 | 4 |
| 25 | Have you made a plan for suicide? | 0 | 1 | 2 | 3 | 4 |
| 26 | Have you ever intentionally hit, pinched, pushed, kicked, or locked someone indoors to hurt them? | 0 | 1 | 2 | 3 | 4 |
| 27 | Do you gamble? | 0 | 1 | 2 | 3 | 4 |
| 28 | Have you ever stolen money? | 0 | 1 | 2 | 3 | 4 |
| 29 | Have you ever driven (bicycle, moped, or car) recklessly (e.g., speeding under the influence of substances)? | 0 | 1 | 2 | 3 | 4 |
| 30 | Have you taken revenge on someone? | 0 | 1 | 2 | 3 | 4 |
| 31 | Have you ever experienced dizziness, cold sweats, physical weakness, or other physical discomfort due to excessive dieting? | 0 | 1 | 2 | 3 | 4 |
| 32 | Have you ever had sexual relations with someone after drinking alcohol or taking certain drugs? | 0 | 1 | 2 | 3 | 4 |
| 33 | Have you ever forcibly demanded property from others? | 0 | 1 | 2 | 3 | 4 |
| 34 | Boys: Have you ever gotten a girl pregnant? Girls: Have you ever been unintentionally pregnant? | 0 | 1 | 2 | 3 | 4 |
| 35 | Have you had sexual relations with 2 or more people? | 0 | 1 | 2 | 3 | 4 |
| 36 | Have you engaged in suicidal behavior (attempted suicide)? | 0 | 1 | 2 | 3 | 4 |
| 37 | Have you carried a weapon (e.g., gun, dagger, wooden stick, etc.)? | 0 | 1 | 2 | 3 | 4 |
| 38 | Have you had sexual relations with someone you didn't know? | 0 | 1 | 2 | 3 | 4 |
