# Supplementary material for: Prevalence and risk factors of depression in college students in Northeast China during the COVID-19 pandemic: a cross-sectional study
Source: BMC Psychol. 2026 Jan 7;14:171. doi: 10.1186/s40359-025-03944-x (PMC12869974; doi:10.1186/s40359-025-03944-x)
Supplement: Supplementary file 4 — Supplementary Material 4. [file 40359_2025_3944_MOESM4_ESM.docx]

****Self-Rating Depression Scale (SDS)****

| **No.** | **Question** | **Little or None** | **Some of the time** | **Good part of time** | **Most of time** |
| --- | --- | --- | --- | --- | --- |
| 1 | I feel down-hearted and blue | 1 | 2 | 3 | 4 |
| 2 | I feel best in the morning | 1 | 2 | 3 | 4 |
| 3 | I have crying spells or feel like it | 1 | 2 | 3 | 4 |
| 4 | I have trouble sleeping at night | 1 | 2 | 3 | 4 |
| 5 | I eat as much as I used to | 1 | 2 | 3 | 4 |
| 6 | I still enjoy sex | 1 | 2 | 3 | 4 |
| 7 | I notice that I am losing weight | 1 | 2 | 3 | 4 |
| 8 | I have trouble with constipation | 1 | 2 | 3 | 4 |
| 9 | My heart beats faster than usual | 1 | 2 | 3 | 4 |
| 10 | I get tired for no reason | 1 | 2 | 3 | 4 |
| 11 | My mind is as clear as it used to be | 1 | 2 | 3 | 4 |
| 12 | I find it easy to do the things I used to | 1 | 2 | 3 | 4 |
| 13 | I am restless and can't keep still | 1 | 2 | 3 | 4 |
| 14 | I feel hopeful about the future | 1 | 2 | 3 | 4 |
| 15 | I am more irritable than usual | 1 | 2 | 3 | 4 |
| 16 | I find it easy to make decisions | 1 | 2 | 3 | 4 |
| 17 | I feel that I am useful and needed | 1 | 2 | 3 | 4 |
| 18 | My life is pretty full | 1 | 2 | 3 | 4 |
| 19 | I feel that others would be better off if I were dead | 1 | 2 | 3 | 4 |
| 20 | I still enjoy the things I used to do | 1 | 2 | 3 | 4 |
