# Supplementary material for: Prevalence and risk factors of depression in college students in Northeast China during the COVID-19 pandemic: a cross-sectional study
Source: BMC Psychol. 2026 Jan 7;14:171. doi: 10.1186/s40359-025-03944-x (PMC12869974; doi:10.1186/s40359-025-03944-x)
Supplement: Supplementary file 5 — Supplementary Material 5. [file 40359_2025_3944_MOESM5_ESM.docx]

****Survey Questionnaire****

Dear students,

Hello! Thank you for participating in this survey. Please read the following instructions carefully before you begin answering the questions:

When answering the questions, please circle "Ο" or check "√" the number that best matches your actual situation.

Please complete the questionnaire independently. Do not discuss the answers with others.

After completing the questionnaire, please check your answers carefully to avoid any mistakes or omissions.

****Date of Completion:**** ____ Year ____ Month ____ Date

****I. Basic Information****

****1. Gender:****
 ① Male
 ② Female

****2. Date of Birth:****
 ____ Year ____ Month ____ Day (Gregorian Calendar)

****3. School:****
 _________________________

****4. Major:****
 _________________________

****5. Grade:****
 ① Freshman
 ② Sophomore

****6. Family Location (Household Registration):****
 ① Rural
 ② Urban or Town

****7. Are you the only child in your family?****
 ① Yes
 ② No → If no, how many children are there in your family? ____
  You are the ____ child (in birth order).

****8. Your family's monthly income is:****
 ① Below 4000 RMB
 ② 4001 - 8000 RMB
 ③ Above 8001 RMB

****9. Father's Education Level:****
 ① Junior high school, primary school, or below
 ② High school (including technical secondary school)
 ③ Bachelor's degree or above (including associate degree)

****10. Father's Occupation is:****
 ① Self-employed, Business, Service industry worker
 ② Farmer
 ③ Professional/Technical personnel
 ④ Government/Institution employee
 ⑤ Corporate employee
 ⑥ Other

****11. Mother's Education Level:****
 ① Junior high school, primary school, or below
 ② High school (including technical secondary school)
 ③ Bachelor's degree or above (including associate degree)

****12. Mother's Occupation is:****
 ① Self-employed, Business, Service industry worker
 ② Farmer
 ③ Professional/Technical personnel
 ④ Government/Institution employee
 ⑤ Corporate employee
 ⑥ Other
